# Working with patients suffering from chronic diseases can be a balancing act for health care professionals - a meta-synthesis of qualitative studies

**DOI:** 10.1186/s12913-019-4826-2

**Published:** 2020-02-10

**Authors:** Heidi Holmen, Marie Hamilton Larsen, Merja Helena Sallinen, Lisbeth Thoresen, Birgitte Ahlsen, Marit Helen Andersen, Christine Råheim Borge, Hedda Eik, Astrid Klopstad Wahl, Anne Marit Mengshoel

**Affiliations:** 1 Faculty of Health Sciences, Department of Nursing and Health Promotion, Oslo Metropolitan University, Oslo, Norway; 2 Faculty of Medicine, Institute of Health and Society, Department of Interdisciplinary Health Sciences, University of Oslo, Box 1089, Blindern, 0317 Oslo, Norway; 3 Lovisenberg Diaconal University College, Oslo, Norway; 4 Faculty of Health and Welfare, Satakunta University of Applied Sciences, Pori, Finland; 5 Faculty of Health Sciences, Department of Physiotherapy, Oslo Metropolitan University, Oslo, Norway; 6 Department of Transplantation Medicine, Oslo University Hospital, Oslo, Norway; 7 Lovisenberg Diaconal Hospital, Oslo, Norway

**Keywords:** Health personnel, Work experiences, Noncommunicable disease (NCD), Clinical encounters, diabetes mellitus, type 2, Pulmonary disease, chronic obstructive (COPD), Chronic kidney disease (CKD), patient-centered care, occupational burden

## Abstract

**Background:**

The number of patients with long-term chronic diseases is increasing. These patients place a strain on health care systems and health care professionals (HCPs). Presently, we aimed to systematically review the literature on HCPs’ experiences working with patients with long-term chronic diseases such as type 2 diabetes, chronic obstructive pulmonary disease (COPD), and chronic kidney disease (CKD).

**Method:**

A systematic search of papers published between 2002 and July 2019 was conducted in the Embase, AMED, PsycINFO, MEDLINE, CINAHL, and COCHRANE databases to identify studies reporting qualitative interviews addressing HCPs’ experiences working with adults with COPD, CKD or type 2 diabetes. An interdisciplinary research group were involved in all phases of the study. With the help of NVivo, extracts of each paper were coded, and codes were compared across papers and refined using translational analysis. Further codes were clustered in categories that in turn formed overarching themes.

**Results:**

Our comprehensive search identified 4170 citations. Of these, 20 papers met our inclusion criteria. Regarding HCPs’ experiences working with patients with COPD, CKD, or type 2 diabetes, we developed 10 sub-categories that formed three overarching main themes of work experiences: 1) individualizing one’s professional approach within the clinical encounter; 2) managing one’s emotions over time; 3) working to maintain professionalism. Overall these three themes suggest that HCPs’ work is a complex balancing act depending on the interaction between patient and professional, reality and professional ideals, and contextual support and managing one’s own emotions.

**Conclusion:**

Few qualitative studies highlighted HCPs’ general working experiences, as they mainly focused on the patients’ experiences or HCPs’ experiences of using particular clinical procedures. This study brings new insights about the complexity embedded in HCPs’ work in terms of weighing different, often contrasting aspects, in order to deliver appropriate practice. Acknowledging, discussing and supporting this complexity can empower HCPs to avoid burning out. Leaders, health organizations, and educational institutions have a particular responsibility to provide HCPs with thorough professional knowledge and systematic support.

**Trial registration:**

PROSPERO number: CRD42019119052.

## Background

With an increasing number of patients with chronic conditions worldwide, a growing number of health care professionals (HCPs) will encounter these patients daily. As patients live longer with chronic diseases, the relationship between HCPs and patients may last for years. In order to provide the best treatment for the growing number of patients with chronic conditions, we need to increase our understanding of how to keep HCPs motivated in their work [1]. This is particularly important today, as burn-out among HCPs is a growing problem [2].

The global increase of chronic diseases, often framed as non-communicable diseases (NCDs), has resulted in global strategies within the World Health Organization (WHO) and the United Nations to prevent or delay onset and reduce premature mortality [3]. Lifestyle choices and unhealthy lifestyle habits, such as physical inactivity, an unhealthy diet, and smoking, are commonly linked to NCDs. If these chronic conditions are diagnosed at an early stage and adequate self-management strategies are applied, the prognosis can be good. Self-management of chronic disease entails an active role for patients, who must make day-to-day decisions to manage symptoms, treatment, physical and psychosocial consequences of the disease, and lifestyle changes [4]. Thus, self-management within long-lasting care is often individualized, goal-oriented, and facilitated in collaboration with HCPs [5]. Nevertheless, these patients will often depend on support from the health care system for the rest of their lives, and the likelihood that these patients will leave the health care service once they have entered is low. Patients that need long-term support thus represent a significant and growing strain on the system and on the involved HCPs.

For the past few decades, patients have been encouraged to take responsibility for their health to a greater degree [6]. This is certainly the case for those living with chronic diseases, as patients are now expected to take an active role in their disease management: e.g. to follow medical regimes and comply with recommendations concerning lifestyle changes and adjustments in their daily lives. This requires that patients have easy access to information that is relevant to their specific situation. However, it may also challenge the HCP’s role, as it represents a shift from a paternalistic approach, in which the HCP is the expert, to an approach in which the patient is acknowledged as an expert on his/her own life, with the right to make informed decisions regarding his/her self-management [6]. Here, providing the tailored information and support for each individual patient at the right time, as well as involving and engaging the patients in their care, is important.

The patient’s expertise is highly emphasized in patient-centred care, which is defined as care “that is respectful of and responsive to individual patient preferences, needs, and values” and that ensures “that patient values guide all clinical decisions” [7]. This definition of patient-centred care highlights the importance of clinicians and patients working together to produce the best possible outcomes. Several researchers are proponents of this concept; however, the question has also been raised how HCPs best should handle situations where a patient makes decisions that worsen disease outcomes [8, 9]. Repeated clinical encounters in which expectations regarding the patient role as expert are not met can contribute to this conflict, and possibly increase the already high strain and expectations placed on HCPs. Furthermore, the new HCP and patient roles are not necessarily thoroughly addressed during professional training. Thus, presumably HCPs must continuously balance their professional expertise with regards to responsibility and decision-making: taking responsibility and making decisions for the patient, or letting the responsibility lie with the patient, who may make decisions that include unhealthy behaviors.

Political statements recommend patient-centered care to a growing population of patients, many with NCDs [10, 11]. At the same time, health care are governed by ideals of public management requesting time-limited and effective care. However, the delivery of patient-centered care may take time and as outlined above - even not be effective in improving a patient’s health outcomes. Hence, dilemmas and challenges may arise for the HCPs that cannot be easily solved in clinical practice [12]. Indeed, in studies of nurses and physicians, burn-out and job dissatisfaction is associated with plans to leave their jobs [2].

Previous research on HCPs’ experiences of long-term patient–provider relationships is scarce, and often related to professional practice procedures rather than to the HCPs’ individual working experiences in general. How to understand the patient’s experiences is widely researched, but how the HCPs experience the patient–provider relationship, particularly when patients have a chronic disease, appears not to have generated equal interest among scientists. One qualitative study aiming to elicit providers’ perspectives on caring for patients with chronic pain found that HCPs internalized feelings of failure, guilt and discontent. This study highlighted the need for physicians to care not just for their patients but also to adopt self-care strategies to reduce “compassion fatigue” when caring for challenging patients [13].

The present systematic review of qualitative empirical studies aims to provide an in-depth insight regarding how HCPs experience working with patients with chronic diseases. We decided to focus on chronic obstructive pulmonary disease (COPD), chronic kidney disease (CKD), and type 2 diabetes, as these share commonality regarding the continuity of HCP–patient interactions over time and the great impact of lifestyle and self-management on prognosis. However, the three diseases also have clear differences in their treatment, their need for HCP follow up and prognosis over time, making them suitable to contrast the HCP experience. Thereby, we sought new insights that could aid HCPs, policymakers, and educational institutions in reducing the strain on HCPs and preventing burn-out among HCPs. Thus, our aim was to systematically review the literature on HCPs’ experiences working with patients with long-term chronic diseases such as type 2 diabetes, COPD, and CKD.

## Methods

### Design

This systematic review was conducted between October 2017 and June 2019. A research group comprising 10 senior researchers (the authors), with a professional background in either nursing or physiotherapy and qualified in realist and interpretive qualitative research methods, conducted a systematic literature review of qualitative papers concerning HCPs’ experiences working with patients with type 2 diabetes, CKD, and COPD. The members of the research group had all worked in clinical and research settings with patients with a range of chronic diseases. Whereas most of the nurses had insider knowledge from their clinical work and research on diabetes, COPD, or CKD, the physiotherapists had an outsider perspective, as their experiences related mainly to chronic musculoskeletal disorders. These differences nurtured our discussions during the whole process. In order to enhance the researcher’s reflexivity, shifting pairs of researchers worked together in all phases of the review process of inclusion/exclusion, appraising the methodological quality, extracting the data for further analysis, and analyzing the data. In the initial phase of the systematic review, a protocol was published in PROSPERO. The review protocol may be accessed via PROSPERO under the registration code CRD42019119052.

### Information sources and search

At beforehand, we decided to include only empirical qualitative studies published in scientific journals, and grey literature, conference proceedings, master and PhD thesis were excluded as they often lack peer reviews. A systematic search strategy was developed and revised in close collaboration among the researchers and with assistance from an experienced research librarian before the final search was conducted. This comprehensive strategy aimed to ensure that relevant peer-reviewed empirical studies were identified in the search. The searches were conducted in six databases: Embase, AMED, PsycINFO, MEDLINE, CINAHL, and COCHRANE. The Medline’s Medical Subject Headings (MESH) terms and additional keywords were used to identify relevant search terms, and the librarian added a study-specific “qualitative filter” to further tailor the search strategy. The search strategy was then adjusted to each other databases. While publication language was not limited, publication dates were limited to between 2002 and 2017. The original search was completed by the 29th of November 2017 and was updated in June 2019 by the same librarian. An example of the search strategy can be found in the Additional file 1.

### Eligibility criteria

We based our search strategy on the “SPIDER” framework—an acronym for sample, phenomenon of interest, design, evaluation, and research type [14]—to identify the eligibility criteria, as shown in Table 1. The SPIDER framework was chosen based on its applicability for qualitative research.

**Table 1:** The SPIDER framework

*Sample (S):*	We included studies addressing the experience of HCPs working with adults with chronic conditions, in which the relationship between the patient and HCP is assumed to be long-term. In this review, we focused on diabetes, COPD, and CKD. If a study included a subset of eligible participants (e.g. a mixed population, including participants with other health conditions), we only included it if we could analyze the disaggregated data for the eligible participants separately. We excluded studies exploring any pre-existing phases of the three selected diseases, e.g. pre-diabetes, and those studies that included children aged 18 and younger.
*Phenomenon of interest (PI):*	We included any empirical, qualitative studies exploring the experiences of the relationship between HCP and patients with the selected chronic conditions reported from the HCPs perspective. We also included studies addressing the HCPs’ feelings, attitudes, and perceptions, work satisfaction, or emotional burden regarding their relationship with these patients over time. We excluded studies addressing other phenomena, such as experiences with patients’ use of specific treatments or interventions and those focusing on palliation.
*Design (D):*	We included studies utilizing empirical qualitative research, including either individual or focus group interviews inspired by ethnography, narrative methods, phenomenology, grounded theory, observations, or qualitative interviews with no specific theoretical statements. We excluded: quantitative designs, mixed methods studies, studies reporting results from both patients and HCPs, studies that did not address HCPs’ experiences working with patients with diabetes, COPD, or CKD, studies exploring the experiences of surgeons, pharmacists, or students (who were assumed to not have a long-term relationship with their patients), and studies that addressed HCPs experiences with specific interventions or treatments.
*Evaluation (E):*	In all the qualitative studies, quotes from interviews of the HCPs had to be reported and analyzed qualitatively in the article for it to be included in the review.
*Research type (R):*	We included all published qualitative research, with no language restrictions. Grey literature, such as conference proceedings and non-peer reviewed articles, were excluded, as they lack quality, detail, and peer review. We also excluded systematic reviews and meta-syntheses, as well as masters and PhD theses.

### Study selection

The final search strategy identified titles and abstracts. In the screening process, all authors participated in pairs, and the papers were independently screened by title and abstract for eligibility by the two reviewers in each pair, before the pairs met and discussed. Each file represented one diagnosis; one pair sorted through the files for COPD, one pair sorted through chronic kidney disease, and three pairs sorted through the files for diabetes. The inclusion and exclusion criteria were developed a priori but refined to become more precise during the inclusion process. The authors discussed each reference for which initial agreement was not reached, before a final set of references were retrieved and reviewed in full-text to assess whether they met the inclusion criteria. The included papers were then read by new pairs within the group to consolidate the agreement across the reviewers.

### Study characteristics

The included papers were presented in a table drafted by the research group. This table included study specifications (author, country, and year of publication), number and gender of participants, characteristics, research purpose, stated theoretical or philosophical perspective, recruitment source, data collection, data analysis, main findings related to our research purpose, and whether the study fulfilled the methodological appraisal.

### Methodological appraisal

To assess the methodological quality of the studies, the Critical Appraisal Skills Programme checklist (CASP) for qualitative research was chosen as it is not nested to a particular epistemological perspective [15]. In addition, one domain from the Consolidated Criteria for Reporting Qualitative research (COREQ) was applied to capture methodological orientation and theory [16]. Working in pairs, the papers were first independently appraised by two researchers, before each pair met and discussed their appraisal, and the results were discussed by the group until consensus was reached about how we interpreted the items and came to a conclusion. Papers of poor quality was not excluded, as papers with poor conceptual development are considered to contribute less to the results [17, 18]. We argue that a methodological appraisal of the included papers is most valuable for describing the methodological quality to inform methodological discussions for future studies.

### Data extraction and analysis

After the inclusion/exclusion process, the authors read all the included papers in full text in order to identify the data at hand. Our general impression from this reading was that HCPs, in general, were satisfied with their work, but the general outline of several challenges could also be seen. These were discussed by the group. This overall impression guided our further (detailed) analysis of each paper, but it also enabled us to critically appraise whether our initial impression was supported by the data.

The detailed, in-depth analysis was performed by groups of two or three members who each independently determined what was relevant in the primary studies’ result section to inform our research question and subsequently to be extracted for further analysis. The extracts were discussed by the pairs until consensus was reached, and thereafter coded manually by the pairs. Metaphors and concepts within the text were identified and used as codes whenever appropriate. We began with papers that we found presenting a richness of information and concepts in accord with what Britten and Pope [19] call conceptual richness which characterize the best papers.

The extracts of the next papers were then examined to see whether they could be translated into the codes we used for the previous paper; if not, new codes were developed. This approach was inspired by translational analysis, and thus involved an analytical transfer of concepts and insights between studies [18, 20]. Through this extensive process, recurrent or shared concepts—and points of similarity (reciprocal translation) and difference (refutational translation) within these concepts—were identified across studies and explicated iteratively. The small groups then presented their decisions for the whole group. As everyone had read all of the papers, the extracts were re-discussed until consensus was reached.

The extracts and codes were transferred into the NVivo software program. Here, too, all extracts and codes were read and discussed by the group and modified by looking across all the coded papers. This process generated 36 codes (“nodes” in NVivo), which were written on Post-It Notes and attached to a whiteboard. The group then shared, reviewed, and discussed the codes, and finally clustered them into categories. Through discussions and critical appraisals based on the group’s mutual knowledge of the data material but various theoretical perspectives, we gradually developed three main overarching themes.

## Results

### Search outcome

**Fig. 1:**
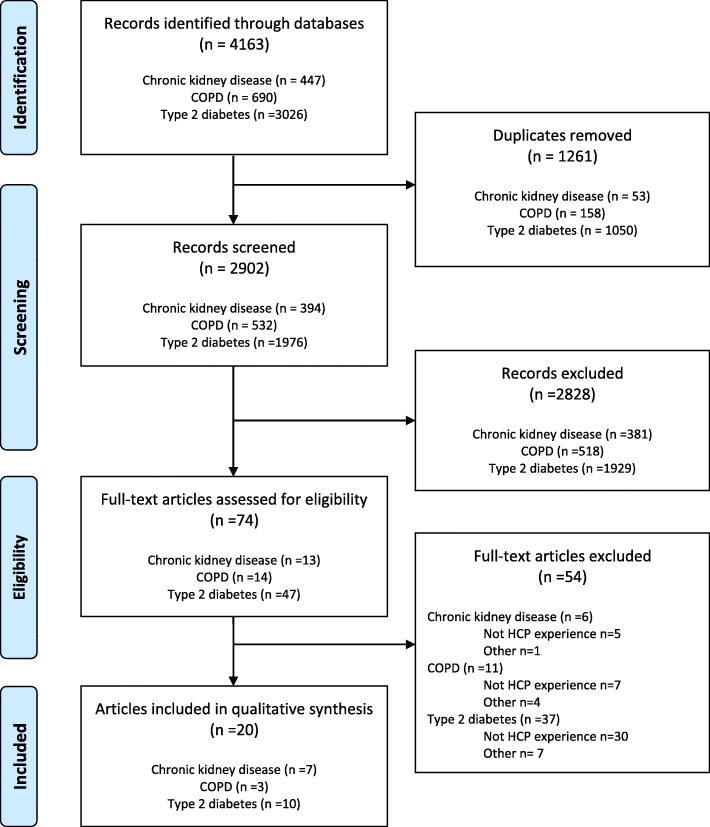
PRISMA Flowchart

The electronic search produced *n* = 4177 references (Fig. 1). Following the abstract review, *n* = 74 publications were selected for full-text review. In total, *n* = 54 publications were excluded during this screening process. The final full-text review produced 20 papers for further analysis.

The included 20 papers concern HCPs’ experiences providing care to patients with CKD (7 papers), COPD (4 papers), or type 2 diabetes (9 papers) (Tables 2 and 3). A total of 456 HCPs participated in the studies; the majority were physicians (*n* = 313) and nurses (*n* = 158). Only two studies included physiotherapists [32, 33], one included a podiatrist [24], and four included dieticians in addition to nurses and physicians [24, 32, 33, 35]. All eligible studies were reported in English, but there was general geographical variation among the studies: 13 were conducted in Europe, 3 were from North America, 2 from Asia, 1 study was from Australia, and 1 study took place in 7 countries in Europe and Asia. As such, the studies cover the experiences of HCPs working in a range of health care systems and cultures. Overall, the methodological quality of the included papers was considered to be high (Table 2 and in detail in Additional file 2). All papers had a clear statement of their research aims and were found to have appropriately applied a qualitative methodology. Further, all papers clearly stated their findings. However, there was a trend across the papers that the researcher’s own role in and impact on the interviews and the analysis was not discussed. Likewise, the studies’ methodological orientation and theoretical framework were rarely described. No studies were excluded from further analysis based on their methodological appraisal.

**Table 2:** Study purpose, theoretical perspective, and quality evaluation results

Study (author, year of publication and country (reference))		Purpose of the papers	The stated theoretical or philosophical perspective	Quality evaluation No. fulfilled items (No. cannot tell)
1	Noor Abdulhadi et al. (2013), Oman [21]	To explore primary health care providers’ experiences working with patients with type 2 diabetes, and their suggestions and preferences regarding future improvements in diabetes care	Not stated	9 (2)
2	Boström et al. (2012), Sweden [22]	To explore diabetes specialist nurses’ perceptions of their professional role in diabetes care	Not stated	9 (2)
3	Brown, Bain, Broderick and Sully (2013), Australia [23]	To identify patterns and themes in how renal nurses and two other nursing specialists engage with patients’ emotional expressions, express their own emotions, and access and provide support for emotional expenditure	Conservation of resources (COR) theory, (Hobfoll, 1989)	9 (2)
4	Craven, Simons and de Groot (2019), USA [24]	To conduct a qualitative exploration of the emotional experiences of healthcare providers engaged in diabetes medical care and describe and understand the unique features of burn-out, as experienced by diabetes providers	Not stated	10 (1)
5	Crawford (2010), UK [25]	To explore HCPs’ level of awareness around COPD patients’ concerns regarding end of life care	Phenomenology	11 (0)
6	Crowshoe et al. (2018), Canada [26]	To describe Canadian physicians’ perspectives on diabetes care of indigenous patients	Not stated	9 (2)
7	Huber et al. (2011), Switzerland [27]	To explore nurses’ perspectives on diabetes care in nursing homes and home health care services, and to describe the existing level of diabetes care in these setting	Not stated	8 (3)
8	Kim et al. (2016), Korea [28]	To understand the lived experience of nurses who care for people undergoing maintenance hemodialysis	Phenomenology; theory of caring (Swanson,1991)	10 (1)
9	Matthews and Trenoweth (2015), UK [29]	To explore nurses’ interpretation of the needs of people with long-term conditions, and their perceptions of subsequent nursing in a renal service	Not stated (self-management?)	6 (5)
10	Pooley, Highfield and Neal (2015), UK [30]	To explore the experience of consultant nephrologists in the long-term doctor–patient relationship	Not stated (phenomenology?)	9 (2)
11	Risør et al. (2013), Norway, Germany, Poland, Wales, Russia, the Netherlands, and China (Hong Kong) [31]	To explore the reasoning of GPs and respiratory physicians when managing patients with COPD exacerbations in clinical encounters	Grounded theory	9 (2)
12	Stuij (2018), the Netherlands [32]	To gain in-depth insight into experiences of health care professionals regarding the delivery of physical activity counseling to patients with type 2 diabetes	Not stated	10 (1)
13	Svenningsson, Hallberg and Gedda (2011), Sweden [33]	To generate a theory grounded in empirical data derived from a deeper understanding of health care professionals’ main concerns when they consult with individuals with both diabetes and obesity and how they handle these concerns	Grounded theory (Glaser and Strauss,1967)	11 (0)
14	Tam-Tham et al. 2016, Canada [34]	To describe barriers, facilitators, and strategies to enhance conservative, non-dialysis CKD care by primary care community physicians working with stage-5 CKD patients	Not stated; COREQ as reporting framework	9 (2)
15	Tierney et al. (2017), UK [35]	To explore compassionate care from the perspective of HCPs working with type 2 diabetes	Compassionate care; grounded theory; constructivism	10 (1)
16	Tonkin-Crine et al. (2015), UK [36]	To explore GPs’ views on managing patients with advanced CKD and their referral to secondary care.	Not stated	10 (1)
17	Walker, Abel, and Meyer (2012), New-Zealand [37]	To describe and discuss what the majority of New Zealand pre-dialysis nurses believe influences their ability to provide effective patient care	Not stated (descriptive exploratory approach)	9 (2)
18	Wens et al. (2005), Belgium [38]	(1) To elicit problems physicians encounter with type 2 diabetes patients’ adherence to treatment recommendations; (2) to search for solutions (3); to discover escape mechanisms in case of frustration	Not stated	9 (2)
19	Wollny et al. (2018) Germany [39]	To reveal GPs’ attitudes of towards type 2 diabetes patients with poor metabolic control	Not stated	10 (1)
20	Zakrisson and Hägglund (2010), Sweden [40]	To describe asthma/COPD nurses’ experience with educating patients with COPD in primary health care	The concept of enablement; the transtheoretical model (TTM)	10 (1)

**Table 3:** Method for data collection and analysis, participants’ characteristics, and main findings

Study authors		Data collection (length of interview) and recruitment method	Sample size and characteristics / Age (mean, range), Gender, Level of experience	Data analysis	Main findings related to the research purpose of the review
1	Noor Abdulhadi et al. [21]	Semi-structured interviews Length: 1 h (on average) Sampling: purposeful sampling + participants from a prior observational study	*N* = 26: 19 doctors, 7 nurses Age: Mean of doctors: 40 years (range: 22–55); mean of nurses: 30 years (range: 25–40) Gender: 15 females, 11 males Level of experience: > 3 years in health care	Qualitative content analysis	Barriers affecting care: 1) work load; 2) frustration with lack of a teamwork approach—doctors perceiving nurses as lacking knowledge and qualifications; 3) poor patient adherence—participants were dissatisfied with the patients’ poor adherence to a healthy diet, exercise and medicines, including refusal of insulin and reluctance to be referred to secondary or tertiary care.
2	Boström et al. [22]	Five semi-structured focus group interviews Length: 50–90 min (median 67 min) Sampling: not recorded	*N* = 29: diabetes nurses Age: mean of 51 years Gender: 27 females, 2 males Level of experience: 15–41 years’ experience working as a nurse; 2–19 years as a diabetes nurse	Qualitative content analysis (Graneheim & Lundman, 2004)	Perceptions of diabetes specialist nurses’ regarding their professional role are presented in five themes: “striving to be an expert,” “striving to be a fosterer,” “striving to be a leader,” “striving to be an executive,” and “striving to be a role model.” Diabetic nursing is a multifaceted profession with roles that cannot be easily combined.
3	Brown, Bain, Broderick and Sully [23]	Semi-structured individual interviews Length: not recorded Sampling: convenience sampling	*N* = 16: 5 renal nurses, 5 emergency nurses, 6 palliative nurses Age: not recorded Gender: 14 females, 2 males Level of experience: 6 months-30 years	Thematic analysis (Dey, 1993).	Renal nurses engage in significant amounts of emotional labor; co-workers are important. They experienced less emotionally confronting situations compared with the two other nursing groups interviewed in the study.
4	Craven, Simons and de Groot [24]	1 focus group (*N* = 5); 5 individual interviews and 13 home-based interviews Length: not recorded Sampling: purposive	*N* = 22: 9 medical residents (primary care physicians and endocrinology fellows), 7 nurses (certified diabetes educators), 4 dietitians, 2 pharmacists Gender: 16 females, 6 males Age: Mean of 43 years Level of experience: average number of years of clinical practice: 13.2 (SD 13.8)	Grounded theory (Corbin & Strauss, 2008)	HCPs reported both positive and negative sides of treating diabetes patients. Several common themes were identified as contributing to distress: patient adherence, negative emotional experiences, emotional fatigue, lack of clear role definition, and work environment concerns. HCPs may experience diabetes-related burn-out.
5	Crawford [25]	In-depth semi-structured face-to-face interviews Length: around 60 min Sampling: purposeful sampling	N = 7: 3 respiratory nurses, 2 lung cancer nurse specialists, 2 respiratory physicians Gender: not recorded Age: not recorded Level of experience: Not recorded, but participants required to have experience communicating with patients at the end of life	Thematic analyses (Edwards & Titchen, 2003)	Anxiety and emotional cost emerged in the face of uncertainty of prognosis and its effects on interactions with patients. The uncertain trajectory increased anxieties for health professionals in initiating discussion. There was a tendency to soften the impact of information given to the COPD patients about death, and HCPs felt unprepared and described anxiety and discomfort.
6	Crowshoe et al. [26]	In-depth semi-structured telephone interviews Length: 1 h Sampling: purposive and convenience sampling	*N* = 28: GPs (3 indigenous family GPs, 21 non-indigenous GPs, 4 diabetes specialists). Gender: 17 males, 11 females Age: not recorded Level of experience: not recorded; (but graduated from medical school between 1970 and 2009)	Thematic analysis and constant comparison analysis using NVivo 9 software	Physicians care were based on humility by acknowledging the limits of their expertise. Feeling guilty not being able to do more. Challenges in building trust, when no continuity of care. Frustrated approximately colleagues not taking into account the sociocultural and political contexts of patients.
7	Huber et al. [27]	4 focus groups Length: 45–60 min Sampling: 4 head nurses recruited participants from among their staff	*N* = 23: nurses Gender: 22 females, 1 male Age: mean of 38 years (range: 23–50) Level of experience: mean of 12.9 years (range: 1–30)	Thematic content analysis	The burden for nurses: lack of information from physicians, low patient acceptance of the disease, caring for elderly patients incapable of decision-making about their care who thus transfer the responsibility to nurses, and varying availability of expertise and levels of competence among the nurses.
8	Kim et al. [28]	Individual in-depth interviews Length: 60–90 min Sampling: purposive sampling	*N* = 14: nurses working at 2 hemodialysis centers Gender: 14 females Age: 33–47 years Level of experience: 8–23 years (with hemodialysis patients: 1.5–18 years, average of 6 years)	Thematic analysis	Nurses were feeling pity for patients and had a continuous efforts to establish a good relationship with the patients. Feeling sadness regarding clients’ lives and lifestyles. Feeling that it is important to make an effort to maintain amicable and therapeutic relationships, but feel burdened by maintaining these relationships in the long term.
9	Matthews and Trenoweth [29]	Individual semi-structured interviews Length: not recorded Sampling: purposive sampling (discontinued due to time restriction)	*N* = 10: staff nurses at the renal ward Gender: not recorded Age: not recorded Level of experience: 6 months-16 years	3-level coding strategy (Corbin and Strauss, 2008)	Nurses experiences high level of responsibility, felt a lack of control and trust in patients’ capacity to self-manage. Experienced stress and anxiety if things go wrong in a patient’s treatment and lack of knowledge and support regarding self-management, lack of time. Threatened by the expert patient.
10	Pooley, Highfield and Neal [30]	Individual semi-structured interviews Length: 33–81 minutesutes (mean: 55 min) Sampling: emails sent to departments nephrologists from the team psychologist	N = 7: nephrologists Gender: 7 males Age: 48 years (mean) Level of experience: mean of 11 years (range: 1–23)	Interpretative phenomaleso-logical analysis (Smith et al., 2009)	Discussing themselves as being more than a doctor, they found the acute scenarios of saving lives the most rewarding aspect. Three main themes: “defining my professional identity,” “relating to the patient,” and “coping with the job.”
11	Risør et al. [31]	21 focus group discussions (FGD). Each country performed 3 FGDs with new participants each time: FGD1—GPs;FGD2—respiratory physicians; FGD3: a mix of GPs and respiratory physicians’ Length: 1–2 h Sampling: purposeful sampling	*N* = 142: urban and rural GPs Gender: not recorded Age: not recorded Level of experience approximately 14 years (50% reported)	Grounded theory, using NVivo	The management of acute COPD exacerbations was handled within a range of concerns, from “dealing with comorbidity” through “having difficult patients” to “confronting a hopeless disease.” Difficulty balancing an approach to a disease that confronts the GP with his professional limits (i.e. concerning curing and saving lives), and with the patient’s existential deterioration at all stages.
12	Stuij [32]	Individual interviews with qualitative and narrative design Length: 30 min to 2 h (average: 1 h) Sampling: purposive in nature.	*N* = 24: 8 physiotherapists, 9 nurses, 2 GPs, 1 internist, 1 dietician, 1 exercise coach, 1 exercise expert, 1 health specialist Gender: 7 males, 17 females Age: mean of 44 years (range: 25–64) Level of experience: average of 15 years (range: 1–40)	Iterative process - aligning with a narrative approach. Data were coded using Max QDA, version 12.0	Two areas of tension regarding physical activity counseling: (1) the understanding of patient behavior; and (2) professionals’ views on responsibilities, including their own (as professionals), and on who is responsible for behavior change. HCPs expressed ambivalent feelings about these themes.
13	Svenningsson, Hallberg & Gedda [33]	7 focus groups and goal 3 individual interviews Length: 30–60 minutes Sampling: initially open, then theoretical	*N* = 20 (13 nurses, four physicians, two dieticians, one physiotherapist) Age: Not recorded Gender: Not recorded Level of experience: > 15 years of working experience	Grounded theory	Ambivalences and uncertainties as to how to coach. Feeling down when failure occurs or there is no change in lifestyle to lose weight. HCPs’ main goal: to give professional individualized care and to find the right strategy for each individual with diabetes and obesity.
14	Tam-Tham et al. [34]	Individual semi-structured telephone interviews Length: 30 min Sampling: purposive sampling (snowball); principle of saturation)	*N* = 27: primary care physicians (PCPs) Gender: 15 males, 12 females Age: < 40: 2; 40–60; 15; > 60: 10 Level of experience: > 20 years: 14; < 10 years: 5; 10–20: 8	Content analysis; reflexive and iterative analysis process	Barriers found were managing patient and family expectations of CKD; challenges associated with managing patients jointly with specialists. Facilitators were to establish patient and family expectations of CKD early; to preserve continuity of care; utilizing a multidisciplinary team approach.
15	Tierney et al. [35]	4 focus groups and 13 interviews (11 by telephone + 2 face-to-face) Length: focus groups: 40–80 min; interviews: 40–75 min Sampling: purposive sampling, snowballing later employed to support theoretical sampling	*N* = 36: 13 nurses, 7 doctors, 6 podiatrists, 5 assistants, 3 dietitians, 2 administrative staff Gender: 29 females, 7 males Age: not recorded Level of experience: 1 month-36 years (with type 2 diabetes)	Constructivist approach (Charmaz, 2014); NVivo used after focused codes were developed	HCPs needed to work in a setting that supported them in their efforts to provide compassionate care. The compassionate care flow could be enhanced by “defenders” (e.g. having supportive colleagues, seeing the patient as a person, drawing on their faith) or depleted by “drainers” (i.e. competing demands on time and resources).
16	Tonkin-Crine et al. [36]	Semi-structured telephone interviews Length: not recorded Sampling: purposive sampling; principle of saturation; 353 UK GPs were invited to participate	*N* = 19: GPs Gender: 12 males, 7 females Age: 46 (31–60) Level of experience Mean years in practice: 16 (range: 3–32)	Inductive thematic analysis, with NVivo	Limited experience with patients led to a lack of confidence managing patients without input from specialists. The difficulty of explaining the diagnosis to patients concerning the asymptomatic nature of CKD. The GPs’ felt managing patients in primary care was preferable and they postponed referrals or felt unsure referring older patients with comorbidities whom they perceived to be unlikely to benefit from dialysis.
17	Walker, Abel & Meyer [37]	Semi-structured telephone interviews Length: approximately 1 h Sampling: purposive sampling	*N* = 11: nurses (almost all pre-dialysis nurses, working in New Zealand) Gender: not recorded Age: not recorded Level of experience: 2–9 years; 6 participants had some form of post-graduate qualification	Thematic analysis and general inductive approach (Thomas, 2006)	Nurses need to have time to provide adequate education and support. Problems with inter-professional relationships and professional autonomy: “role trouble” with regards to making decisions for patients, a lack of facilities and a lack of support from doctors. Difficulty getting promoted to nurse practitioner role and feeling excluded from planning on a strategic level.
18	Wens et al. [38]	Focus group interviews Length: < 2 h Sampling: purposeful sampling	*N* = 40: GPs Gender: 26 males, 14 females Age: mean of 45.3 years (SD 10.5) Level of experience Mean years of practice: 18.4 (SD 10.3)	Content analysis	GPs may get angry when they think the patients do not appreciate their expertise. Frustration leads to a paternalistic attitude. GPs often go along with the patients’ complaints and questions and miss a more structured approach to diabetes. The GPs often feel they have too little time to give detailed advice or explanations.
19	Wollny et al. [39]	In-depth narrative interviews Length: 28 to 80 min (mean: 47 min) Sampling: randomly selected GPs from a larger mixed methods study	N = 20: GPs Gender: 14 males, six females Age: mean of 53.5 years (SD 7.2) Level of experience: mean years of practice: 17.3 (SD 6.6)	Conventional (i.e. inductive) content analysis	GPs feel personally affected by conflicts with their patients. Unable to reach their aims, they suffer from feelings of failure and defeat. The GPs claim to know what is best for their patients but have a difficult time to understand why their advice is not being followed.
20	Zakrisson and Hägglund [40]	Individual interviews, consisting of narratives about nurses’ experiences educating patients with COPD Length: 20–30 min Sampling: not recorded	*N* = 12: asthma/COPD nurses, 8 had specialist education in asthma or COPD at university levelGender: not recorded Level of experience: median: 7 years (with asthma/COPD)	Qualitative content analysis method (Graneheim and Lundman, 2004)	Asthma/COPD nurses’ experience of patient education fluctuated between insecurity and security. Nurses need the support of colleagues and management and more knowledge on patient education methods to be secure. The feeling of being important to the patient is important.

*GP* general practitioner, *CKD* chronic kidney disease, *HCP* health care professionals, *Nvivo* software for organizing categorize and classify data from qualitative and mixed-methods data

### Synthesis of findings

Based on our analysis of the results chapters of the included studies, three main themes were identified and developed, each addressing our overall aim to describe HCPs’ experiences working with patients with long-term chronic diseases: individualizing the professional approach within the clinical encounter; managing one’s emotions over time; and working to maintain professionalism (Table 4). Below, these themes are presented in detail.

**Table 4:** Overview of the themes and categories

Individualizing the professional approach within the clinical encounter			
Engaging with a patient as a person	Encountering the chronic condition	Facilitating a shared understanding of the chronic condition	
Managing one’s emotions over time			
The challenges connected to a long-term relationship	Maintaining professional sympathy	Burden of responsibility	
Working to maintain professionalism			
Striving to achieve the best for the patient	Collaborating with other professionals	Keeping up professional self-esteem	Adjusting to health organizational structures

### Individualizing the professional approach within the clinical encounter

This theme comprises three categories illuminating the clinical encounter between the HCP and the patient: “engaging with the patient as a person,” “encountering the chronic condition,” and “facilitating a shared understanding of the chronic condition.”

The first category, “engaging with the patient as a person,” describes how many HCPs sought to provide individualized care and find ways to approach each patient’s specific needs [27, 33]. As Pooley et al. [30] stated, for HCPs, this entailed getting to know the patient as a person. It was not necessarily easy for HCPs to provide individualized care and recognize the person behind the diagnosis. HCPs tolerated being given inaccurate information from their patients, in order to establish and maintain contact and build positive relationships with them [28, 37]. Other HCPs, in contrast, rarely asked for personal details from their patients, as they did not see the need to do so [21, 29]. In this way, they rejected the idea of tailoring their care based on information provided by their patients.

The second category refers to that, although the HCPs emphasized the importance of seeing and engaging in “the person behind the diagnosis,” they were also obligated to “encounter the chronic condition.” As such, they had to weigh the personal aspects against their professional responsibility and expertise concerning the disease. One challenge for the HCPs, as shown in Tonkin-Crine et al. [36], was the discrepancy between the patient’s subjective experiences on the one hand and clinical measures of the disease on the other. For example, it was a challenge to advocate diet adjustments to patients with kidney failure who had not yet experienced symptoms [36]. It was also challenging for HCPs when patients resisted modifying their lifestyle to optimize the treatment effects [21, 34], for example making decisions that might worsen symptoms and speed its progression.

Further, the HCPs described feeling responsible for ensuring and strengthening patients’ understanding of their disease in order to motivate them to make the recommended lifestyle changes [22]. Despite such efforts, the HCPs felt that some patients showed a lack of interest in self-management, and they considered these patients to be passive and dependent on their HCP. Doctors were especially frustrated with patients who demonstrated low adherence to prescribed treatments [31], and they felt that recommendations were more likely to be poorly followed by the elderly and less-educated [21]. Overall, success was perceived as challenging to predict, if it was even possible to predict [33]. Further, Boström et al. pointed out that efforts to motivate patients to self-manage their condition were perceived as time-consuming for the staff and therefore not always prioritized [22].

The third category, “facilitating a shared understanding of the chronic condition” among HCPs and patients,” reflects that long-term chronic diseases lack curative treatment, and much depends on self-management efforts of patients. A lack of understanding among family members may negatively influence the patient’s acceptance of a disease and its severity [38]. Repeated encounters with patients and next of kin who were unwilling or unable to recognize the severity of the disease prognosis seemed to cause hopelessness among HCPs [31]. The HCPs requested educational tools to assist them in facilitating patients’ and family members’ understanding and acceptance [31, 34]. Some of the HCPs recommended that any change in behavior or life style, however small, to be acknowledged by HCPs to keep their patients motivated [33].

Patients’ specific cultural contexts could also impact their understanding of the chronic disease, which HCPs had to take into account in clinical encounters. This was especially clear in the papers addressing HCPs’ experiences working with ethnic minority groups and indigenous patients [26, 33]. Ethnic minorities were perceived by HCPs as more difficult to reach and cooperate with than most other patients. Here, HCPs also expressed challenges related to their own and colleagues’ lack of knowledge and cultural competence [26]. In particular, conducting consultations in which the HCP was dependent upon a translator complicated the patient–provider relationship [40]. Thus, reaching a mutual understanding of the disease was often a demanding process, because patients often trusted their cultural traditions more than the HCPs’ explanations. Here, lifestyle changes related to decreasing or omitting the consumption of traditional foods was especially difficult [21].

### Managing one’s emotions over time

The theme “managing one’s emotions over time” encompasses the following three categories: “the challenges connected to a long-term relationship”; “maintaining professional sympathy,” and “burden of responsibility.”

The category “the challenges connected to a long-term relationship” includes both the challenges and the rewards associated with long-term patient–provider relationships. With respect to chronic diseases, HCPs often follow the same patients over a long period of time, often spanning years. Pooley et al. [30] argued that doctors must be prepared to become responsible for managing the care of some patients for the rest of their careers. Personal relationships with patients were often developed and appreciated—as shown for example in a study on renal nurses—and these relationships often felt similar to those HCPs had with their friends and family [23].

The HCPs felt that they had to engage emotionally with their patients, and they largely regarded this as positive. However, a long-term patient–provider relationship could also lead to negative personal relationships with patients [24]. If the relationship was poor, it could become challenging for HCPs to provide individualized care. Nurses working with hemodialysis handled this by maintaining a professional and emotional distance from their patients, for example, by simply accepting the patients’ demands; others felt pride when they successfully managed the more difficult relationships [28]. In some cases, HCPs decided to transfer the responsibility for the treatment of a patient to a colleague [23]. In others, HCPs stayed in the struggle, and ultimately developed a better understanding of why the patients appeared to be so difficult [35]. Accordingly, clinical encounters could cause stress, frustration, and tension among the HCPs. However, there were also positive aspects of having personal relationships with patients. One example from a renal ward highlights how doctors valued their long-term, friendly relationships with patients throughout the process of renal failure and dialysis, in which they could share their patients’ joy when conditions improved: for example, when a kidney transplantation was successful [30]. However, patients could also die, and if the HCPs had established a positive relationship with their patients, they mourned their loss. This was made more challenging by the fact that their professional role dictated that they push their feelings aside, to better support the families and other patients who had been close to the deceased [23].

The category “maintaining professional sympathy” centers around how HCPs deemed emotional engagement, in the forms of professional sympathy and compassion, as necessary for continuing in their work [25]*.* Nevertheless, there was also a risk that this compassion would be diminished. Over time, HCPs could become desensitized to others, and dehumanization could begin as early as, for example, medical school [35]. There was also a risk that providing patient care based on routine would be prioritized over providing individualized care. Despite routine and dehumanized care, when patients were unstable or did not follow advice, HCPs expressed a variety of emotions, including sadness, powerlessness, aggression, sympathy, frustration, and irritation. Even the most experienced HCPs reported feelings of guilt when they were unable to make patients follow their therapeutic recommendations [33]. When the emotional engagement with their patients became overwhelming, it was a challenge for HCPs to keep their frustrations from spilling over into their relationships with their patients—this was especially true when HCPs were faced with patients with limited coping abilities or those who complained over minor issues [35].

Under the category “burden of responsibility” the HCPs felt highly responsible for their patients’ wellbeing, which they sometimes also experienced as a burden [33]. This was especially true when patients did not follow the treatment recommendations provided by the HCP and their disease worsened. Boström et al. [22] found, for example, that while nurses valued even the smallest behavioral changes accomplished by their patients, they had to accept that, in some cases, it was likely that change would never occur. Further, the nurses described the challenge of balancing their desire to help their patients while also acknowledging that the patients had to help themselves [22]. The HCPs felt highly responsible for their patients, and this frequently resulted in feelings of guilt and shame, even for the most experienced HCPs [33].

### Working to maintain professionalism

While the papers addressed the experiences of HCPs from a range of cultural contexts, working with three different chronic diseases, the HCPs shared the opinion that maintaining professionalism was important. The work maintaining professionals contains the following four categories: “striving to achieve the best for the patient,” “collaborating with other professionals,” “keeping up professional self-esteem,” and “adjusting to health organizational structures.”

The category “striving to achieve the best for the patient” includes HCPs’ experiences working to meet ideals embedded in their professional role and practice context [32, 39]. One goal was to establish positive relationships with the patients, gaining the patients’ trust while also being decisive, flexible, capable, and qualified in their work. Even experienced nurses expressed a fear of being rejected by their patients [28]. Renal nurses were uncomfortable being closely observed by other patients in the dialysis wards where nurses’ professional activities with one patient could be easily observed by other patients [28]. Having a positive relationship with patients was seen as especially important when doctors had to break the news to them about a worsening of their condition [30]. Positive relationships with their patients also involved the patients’ gratitude when their treatments were successful, which generated strong feelings of professional pride and personal satisfaction for the HCPs. In particular, achieving success in acute situations enabled doctors to maintain a positive attitude towards patients requiring long-term chronic care [30].

Walker et al. [37] reported that dialysis nurses found it important to have professional autonomy, as it helped them better manage patients and tailor their care. A lack of professional autonomy, in turn, made providing care more difficult and less effective; however, the nurses felt that the hierarchical system in which they worked constrained their professional autonomy, making them feel they occupied a subordinate position within the hospital hierarchy. Some nurses were afraid to express their opinions and bring up critical issues related to their work [37]. Abdulhadi [21] remarked that a heavy workload, lack of teamwork, and lack of support from superiors in a hierarchical health care system decreased HCPs’ trust in the system: doctors, in particular, reported feeling a lack of trust in their co-workers’ competence. Boström et al. [22] showed how diabetes specialist nurses found their autonomy and self-determination hampered by being frequently told to assist others in their work.

The category we labelled “collaborating with other professionals” encompasses multidisciplinary support and cooperation with colleagues, as well as the professional loneliness that arises from a lack of cooperation and support. On the one hand, colleagues were described as the most prominent source of support, mainly because they knew the context of care and were easily available for consultation [23]. This support could take different forms, through professional discussions in multidisciplinary teams or on a one-to-one basis, and was, if successful, based on a mutual respect for one another and a shared understanding of the challenges to be solved. Receiving support from colleagues was described as stimulating [40], enabling HCPs to take on more responsibilities [37]. Supportive colleagues also contributed positively to the HCPs’ workflow because they provided a space for HCPs to vent or seek advice after a difficult consultation [35]. Furthermore, several studies [36, 37, 40] highlighted the value of receiving acknowledgement and support from superiors. Nurses described needing support and acknowledgement from the physicians, and to some extent from the physiotherapists, while physicians described needing support from medical specialists or leaders within the health context.

On the other hand, the absence of support was also highlighted in the included articles, and could result in feelings of professional loneliness. Nurses, in particular, described feeling left alone with extensive responsibility [40]. Also, general practitioners felt they lacked the confidence to approach their more complex patients without the support of a medical specialist [36]. Professional collaboration was also dependent on the clinical context and the behaviors of the HCPs’ colleagues. For example, in cases where physicians did not treat their patients properly, this had a negative impact on the nurses’ own work [22, 40].

The third category represents “keeping up professional self-esteem.” Positive feedback from others strengthened the HCPs’ self-esteem, and being liked, respected, and valued by colleagues and those in charge in the ward were highlighted as important [30]. Positive patient outcomes also increased positive feelings. For nurses, this signified that their efforts in caring for their patients had an impact [28]. Feeling liked by one’s patients also contributed to enhanced professional self-esteem. In one study, nurses reported that when they spent more time with patients than physicians, they had higher chances of being liked by their patients [40]. In another, asthma and COPD nurses described how acting autonomously enabled them to reach their full potential [40]. However, the nurses also felt that their professional self-esteem decreased when they experienced professionally loneliness, subordination, and a lack of appreciation from their colleagues [22].

The fourth category was “adjusting to health organizational structures,” and this was connected to nurses’ professional development and career possibilities. Although specialized nursing skills were emphasized within a health organization, it was not always considered essential to support the nurses’ goals of learning and mastering these skills [22]. Further, time—the lack of time, in particular—was a structural factor in the health system mentioned by HCPs in several studies [22, 37, 40]. Recognizing the whole person and delivering individualized care was perceived as a time-consuming task [22]; the lack of time was thus seen by HCPs as a threat to the quality of patient care. According to Tierney [35], however, the HCPs’ compassion for their patients was not affected by time-limited encounters.

## Discussion

The HCPs’ experiences working with patients with type 2 diabetes, CKD, or COPD presented in the papers included in our systematic literature review are captured by three themes: “individualizing the professional approach within the clinical encounter,” “managing one’s emotions over time,” and “working to maintain professionalism.” Together, these themes describe how clinical encounters with patients depend, to a large degree, on the personal interaction between patient and professional, real clinical practice situations and professional ideals, and contextual support and managing one’s own emotions. In more general terms, this means that clinical working experiences are constituted by the interactions between persons, contexts, organizational structures, and health policy claims. Despite the diversity in the efficiency of interventions, treatment options, and long-term prognosis of type 2 diabetes, COPD, and CKD, in general the HCPs’ working experiences seem to be rather shared.

Today, health authorities are governed by ideals such as the patients’ right to receive the best evidenced-based care and the efficiency and cost-effectiveness of the service delivery [41]. At the same time, the democratization of health care services has led to increasing user and patient involvement in all stages of illness, including when the patient is diagnosed with a chronic disease [6, 41]. The patient-centered model of care is part of the user involvement movement and has gained attraction in Western societies. This is meant to counteract the paternalism embedded in the clinicians’ traditional role as sole expert, and to support patients in being active agents regarding the health issues that concern their own lives [6]. However, how patient-centered care should be practiced within the context of the public management and evidence-based practice ideals is rarely, if ever, addressed by research. Our analysis suggests that this balancing act can be complex for HCPs and can create several challenges. This is in line with Holen and Kamp [41], who discuss how user involvement has transformed the relationship between patients and HCPs, and how HCPs today face new dilemmas and challenges. Of particular interest, and in keeping with findings from our study, the authors describe how in long-term patient–provider relationships—e.g. between COPD patients and nurses—“new” professionalism contains relational, emotional and pedagogical aspects to motivate and coach patients. The main aim of this “new” approach is to support the patient in taking responsibility for and self-managing his/her health in a time-efficient way. However, dilemma raises if for example patients need time to understand what a disease implies for their way of living.

As the present findings highlight, on the one hand, the patients’ wishes and autonomy must be respected, as they should be considered experts on their own lives, but on the other, patients do not always understand the severity of their disease-related risks, nor do they necessarily make rational decisions. HCPs feel they lack the tools or ability to help patients and family members understand the risks of not following the prescribed treatment plan. In this way, HCPs feel they are to blame by not fulfilling their professional responsibility. This can be particularly challenging when a patient presents a desire for treatment that differs from evidence-based recommendations for best practice. In such cases, HCPs must balance their acting according to patient-centered care and evidenced-based practice models. “Giving up” one’s professional expertise and instead taking on the role of “partner, colleague or co-worker” may be a solution, as described by Alm Andersen [6]. How precisely to balance such incompatible roles needs broad debate in our society. We argue that this is too heavy burden to be placed on and solved by individual HCPs.

For HCPs, a long-term patient–provider relationship can be both a rewarding and disheartening experience. They may establish a kind of friendship with their patients, and hold significance in their patients’ lives as the one person who shares and can make sense of patients’ disease experiences. As our analysis indicates, this can also be a balancing act, as HCPs know that just as the progression of a disease is uncertain, so is the outcome. HCPs must therefore find the balance between personal closeness to and professional distance from patients, to protect their own emotional vulnerability and maintain a supportive professional role for the patients and their families when patients’ conditions deteriorate, or life ends. There is no formula for how to perform this balancing act in practice, as it is usually individually and situationally determined.

In discussing burn-out among HCPs, Dyrbye et al. [2] point to emotional exhaustion and frequent depersonalization as aspects of burn-out. We found traces of these aspects in our study and agree with the authors that more research to understand and improve the work lives and wellbeing of HCPs is needed. Our findings do indicate, however, that supportive colleagues and leaders, as well as acknowledgement and support from leaders within the health organization, can be helpful. This approach can help meet HCPs’ support needs, and empower them—in various ways and at different levels—to remain in highly complex work situations. We also argue that educational institutions have the responsibility to prepare future HCPs to meet this complexity of clinical practice. As far as we know, this issue is largely absent in today’s curricula.

The contribution and trustworthiness of our meta-synthesis depends on the quality of the original publications on the one hand, and on the rigor of our own methodological process on the other. One strength of this meta-synthesis is that we conducted comprehensive searches of six databases, and thereby generated a variety of data to be analyzed. By choosing three different chronic diseases on which to focus, we succeeded to find richness and variability in HCPs’ detailed working experiences. Furthermore, the inclusion and exclusion criteria and search terms were decided in advance of the literature search, and the searches were conducted by an experienced librarian. Another strength of our meta-synthesis is that the study selection, study appraisals, extractions of data in primary studies to be analyzed and coding were performed independently by pairs of researchers, and the use of NVivo further ensured a rigorous and systematic process. In this part of the process, we followed procedures to ensure reliability in line with a realist perspective [42]. In the analysis, however, we followed an interpretive approach inspired by meta-ethnography and a constructivist stance [42]. This enabled us to take advantage of the various theoretical competencies in the research group. The credibility of the analysis depends on how transparent we present our analysis. We have attempted to describe our data material in the result section as close as possible and with reference to the original papers (second order data). Our overall interpretation of the data (third order level) is presented in the discussion in order to make the correspondence between the descriptions in our result section and our further interpretations transparent. We argue that our shifts between rigorous methodological approach and reflexivity based on our various perspectives and understandings during the whole process have strengthened the trustworthiness of this study. Therefore, it is likely that it is a balancing act to work with diabetes-1, COPM and CKD. Furthermore, we think the complexity and dilemmas raised in these studies are likely to be transferable to the work with other chronic diseases as well.

The present study has some limitations that must be noted. Firstly, according to the quality appraisal, as assessed by the CASP and COREQ, the quality of the original articles can be considered high. However, wider methodological orientations or broader philosophical backgrounds were rarely presented or discussed in the included papers. It is also possible that the assumptions made by the authors of the original studies were continued in the meta-synthesis. Secondly, it is noteworthy that although we welcomed both interview studies and observational studies in the meta-synthesis, no observational studies met our inclusion criteria. This implies a knowledge gap, as the HCP–patient relationship and their interactions require research from different vantage points. Thus, further research to examine how clinical practice is performed and contextualized is needed.

## Conclusion

It is clear from this systematic literature review that HCPs’ experience profound stress in their work with patients with COPD, CKD, or type 2 diabetes. On the other hand, they also experience the creation and maintenance of long-term relationships with patients with chronic conditions as personally and professionally rewarding. As such, HCPs must find the balance between personal closeness to and professional distance from patients. They must also balance providing patient-centered care whilst simultaneously developing and strengthening their professional expertise. This underscores the importance, for HCPs, of having systematic support from colleagues, leaders, educational institutions, and health organizations.

## Supplementary information

**Additional file 1.** Search terms.

**Additional file 2.** Detailed study appraisal.

## Data Availability

Please contact MHL.

## Abbreviations

CASP: Critical Appraisal Skills Programme checklist
CKD: Chronic kidney disease
COPD: Chronic obstructive pulmonary disease
COREQ: Consolidated Criteria for Reporting Qualitative research
HCP: Healthcare personnel
MeSH: Medical subject headings
NCD: Non-communicable diseases
PROSPERO: Prospective Register of Systematic Reviews
SPIDER: Sample, phenomenon of interest, design, evaluation, and research type
WHO: World Health Organization;

## References

[1] WHO (2006). The World Health Report 2006 - working together for health.

[2] Dyrbye LN, Shanafelt TD, Sinsky CA, Bhatt J, Ommaya A, West CP, et al. Burnout among health care professionals. A call to explore and adress this underrecognized threat to safe, high-quality care. Natl Acad Med Perspect Expert Voices Health Health Care. 2017. doi: 10.31478/201707b

[3] WHO. Global action plan for the prevention and control of noncommunicable diseases 2013-2020. Geneva: World Health Organization; 2013.

[4] Bodenheimer T, Wagner EH, Grumbach K (2002). Improving primary care for patients with chronic illness. JAMA.

[5] Lorig Kate R., Ritter Philip, Stewart Anita L., Sobel David S., William Brown Byron, Bandura Albert, Gonzalez Virginia M., Laurent Diana D., Holman Halsted R. (2001). Chronic Disease Self-Management Program. Medical Care.

[6] Alm Andreassen T (2018). Service user involvement and repositioning of health care professionals: A framework for examining implications of different forms of involvement. Nordic Welfare Res.

[7] Baker A (2001). Crossing the Quality Chasm: A new health system for the 21st century. BMJ.

[8] Lo MC (2010). Cultural brokerage: Creating linkages between voices of lifeworld and medicine in cross-cultural clinical settings. Health (London).

[9] De Valck C, Bensing J, Bruynooghe R, Batenburg V (2001). Cure-oriented versus care-oriented attitudes in medicine. Patient Educ Couns.

[10] Beaglehole R, Bonita R, Horton R, Adams C, Alleyne G, Asaria P (2011). Priority actions for the non-communicable disease crisis. Lancet.

[11] Hower KI, Vennedey V, Hillen HA, Kuntz L, Stock S, Pfaff H (2019). Implementation of patient-centred care: which organisational determinants matter from decision maker's perspective? Results from a qualitative interview study across various health and social care organisations. BMJ Open.

[12] Sandelowsky H, Hylander I, Krakau I, Modin S, Stallberg B, Nager A (2016). Time pressured deprioritization of COPD in primary care: a qualitative study. Scand J Prim Health Care.

[13] Matthias MS, Parpart AL, Nyland KA, Huffman MA, Stubbs DL, Sargent C (2010). The patient-provider relationship in chronic pain care: providers' perspectives. Pain Med.

[14] Cooke A, Smith D, Booth A (2012). Beyond PICO: The SPIDER Tool for Qualitative Evidence Synthesis. Qual Health Res.

[15] Critical Appraisal Skills Programme. CASP Qualitative Checklist 2018 [Available from: https://casp-uk.net/wp-content/uploads/2018/03/CASP-Qualitative-Checklist-2018_fillable_form.pdf. Accessed 23 Mar 2018.

[16] Tong A, Sainsbury P, Craig J (2007). Consolidated criteria for reporting qualitative research (COREQ): a 32-item checklist for interviews and focus groups. Internat J Qual Health Care.

[17] Campbell R, Pound P, Morgan M, Daker-White G, Britten N, Pill R (2011). Evaluating meta-ethnography: systematic analysis and synthesis of qualitative research. Health Techn Assess.

[18] Noblit GW, Hare RD (1988). Meta-ethnography: synthesizing qualitative studies.

[19] Britten N, Pope C, Hannes K, Lockwood C (2012). Medicine taking for asthma: a worked example of meta-ethnography. Synthesizing qualitative research.

[20] Britten N (2011). Qualitative research on health communication: what can it contribute?. Pat Educ Couns.

[21] Noor Abdulhadi NM, Al-Shafaee MA, Wahlström R, Hjelm K (2013). Doctors’ and nurses’ views on patient care for type 2 diabetes: an interview study in primary health care in Oman. Prim Health Care Res Develop.

[22] Boström E, Isaksson U, Lundman B, Sjölander AE, Hörnsten Å (2012). Diabetes specialist nurses' perceptions of their multifaceted role. Eur Diab Nurs.

[23] Brown S, Bain P, Broderick P, Sully M (2013). Emotional effort and perceived support in renal nursing: a comparative interview study. J Ren Care.

[24] Craven M, Simons Z, de Groot M (2019). Diabetes distress among healthcare providers: a qualitative study. Diab Res Clin Pract.

[25] Crawford A (2010). Respiratory practitioners' experience of end-of-life discussions in COPD. Br J Nurs.

[26] Crowshoe LL, Henderson RI, Green ME, Jacklin KM, Walker LM, Calam B (2018). Exploring Canadian Physicians' experiences with type 2 diabetes Care for Adult Indigenous Patients. Can J Diab.

[27] Huber C, Huber J, Shaha M (2011). Diabetes care of dependent older adults: an exploratory study of nurses' perspectives. Europ Diab Nurs.

[28] Kim S, Lee HZ, Hwang E, Song J, Kwon HJ, Choe K (2016). Lived experience of Korean nurses caring for patients on maintenance haemodialysis. J Clin Nursing.

[29] Matthews T, Trenoweth S (2015). Nurses' perceptions of self-management in renal care. Br J Nurs.

[30] Pooley HM, Highfield J, Neal A (2015). The experience of the long-term doctor-patient relationship in consultant nephrologists. J Renal Care.

[31] Risør Mette Bech, Spigt Mark, Iversen R, Godycki-Cwirko M, Francis N, Altiner A, Andreeva E, Kung K, Melbye H (2013). The complexity of managing COPD exacerbations: a grounded theory study of European general practice. BMJ Open.

[32] Stuij M (2018). ‘Physical activity, that’s a tricky subject.’ Experiences of health care professionals with physical activity in type 2 diabetes care. BMC Health Serv Res.

[33] Svenningsson Irene, R-M. Hallberg Lillemor, Gedda Birgitta (2011). Health care professionals meeting with individuals with Type 2 diabetes and obesity: Balancing coaching and caution. International Journal of Qualitative Studies on Health and Well-being.

[34] Tam-Tham H, Hemmelgarn BR, Campbell DJ, Thomas CM, Fruetel K, Quinn RR (2016). Primary care physicians' perceived barriers, facilitators and strategies to enhance conservative care for older adults with chronic kidney disease: a qualitative descriptive study. Nephrol Dial Transplant.

[35] Tierney S, Seers K, Tutton E, Reeve J (2017). Enabling the flow of compassionate care: a grounded theory study. BMC Health Serv Res.

[36] Tonkin-Crine S, Santer M, Leydon GM, Murtagh FE, Farrington K, Caskey F (2015). GPs' views on managing advanced chronic kidney disease in primary care: a qualitative study. Br J Gen Pract.

[37] Walker R, Abel S, Meyer A (2012). Perceptions of key influences on effective pre-dialysis nursing care. Contemp Nurs.

[38] Wens J, Vermeire E, Royen PV, Sabbe B, Denekens J (2005). GPs' perspectives of type 2 diabetes patients' adherence to treatment: a qualitative analysis of barriers and solutions. BMC Fam Pract.

[39] Wollny A, Pentzek M, Herber OR, Abholz HH, In der Schmitten J, Icks A (2018). General practitioners' attitudes towards patients with poorly controlled type 2 diabetes: a qualitative study. BMC Fam Pract.

[40] Zakrisson AB, Hagglund D (2010). The asthma/COPD nurses' experience of educating patients with chronic obstructive pulmonary disease in primary health care. Scand J Caring Sci.

[41] Holen M, Kamp A (2018). Brugerinddragelse - ny professionalisme og nye omsorgsrum?. Nordic Welfare Res.

[42] Tong A, Flemming K, McInnes E, Oliver S, Craig J (2012). Enhancing transparency in reporting the synthesis of qualitative research: ENTREQ. BMC Med Res Methodol.

