# Supplementary material for: Working with patients suffering from chronic diseases can be a balancing act for health care professionals - a meta-synthesis of qualitative studies
Source: BMC Health Serv Res. 2020 Feb 10;20:98. doi: 10.1186/s12913-019-4826-2 (PMC7011477; doi:10.1186/s12913-019-4826-2)
Supplement: Supplementary file 1 — Additional file 1. Search terms. [file 12913_2019_4826_MOESM1_ESM.docx]

| Database(s): **Ovid MEDLINE(R)** | |
| --- | --- |
| Search Strategy: | |
| **#** | **Searches** |
| 1 | exp Attitude of Health Personnel/ |
| 2 | exp Professional-Patient Relations/ |
| 3 | exp Burnout, professional/ |
| 4 | ((burnout or burn-out) adj2 professional*).tw,kw. |
| 5 | 1 or 2 or 3 or 4 |
| 6 | exp Adaptation, Psychological/ |
| 7 | exp Emotions/ |
| 8 | exp Stress, Psychological/ |
| 9 | exp Decision Making/ |
| 10 | Job Satisfaction/ |
| 11 | Occupational Diseases/ |
| 12 | exp Sleep Wake Disorders/ |
| 13 | exp Fatigue/ |
| 14 | Personal Satisfaction/ |
| 15 | exp Behavioral Symptoms/ |
| 16 | 6 or 7 or 8 or 9 or 10 or 11 or 12 or 13 or 14 or 15 |
| 17 | (((moral or psychological) adj3 (distress or stress)) or (long adj2 relation*) or burnout or burn-out or emotion* or avoidance* or uncertain* or satisfaction or satisfie* or fatigue* or empath* or exhaust* or fulfil* or content* or experience*).tw,kw. |
| 18 | exp Medical staff/ |
| 19 | exp Nurses/ |
| 20 | exp Nursing staff/ |
| 21 | Nutritionists/ |
| 22 | Occupational therapists/ |
| 23 | Physical Therapists/ |
| 24 | exp Physicians/ |
| 25 | Social Workers/ |
| 26 | (nurse* or physician* or doctor* or (general adj2 practitioner*) or GP* or psychologist* or psychotherapist* or dietician* or dietitian* or nutritionist* or (occupational adj2 therapist*) or (physical adj2 therapist*) or physiotherapist* or (social adj2 worker*) or (health adj2 (personnel or professional* or worker*))).tw,kw. |
| 27 | 18 or 19 or 20 or 21 or 22 or 23 or 24 or 25 |
| 28 | 16 and 27 |
| 29 | ((((moral or psychological) adj3 (distress or stress)) or (long adj2 relation*) or burnout or burn-out or emotion* or avoidance* or uncertain* or satisfaction or satisfie* or fatigue* or empath* or exhaust* or fulfil* or content* or experience*) adj3 (nurse* or physician* or doctor* or (general adj2 practitioner*) or GP* or psychologist* or psychotherapist* or dietician* or dietitian* or nutritionist* or (occupational adj2 therapist*) or (physical adj2 therapist*) or physiotherapist* or (social adj2 worker*) or (health adj2 (personnel or professional* or worker*)))).tw,kw. |
| 30 | 5 or 28 or 29 |
| 31 | exp Qualitative Research/ |
| 32 | Focus Groups/ |
| 33 | interview/ |
| 34 | Interviews as Topic/ |
| 35 | anecdotes as topic/ |
| 36 | Narration/ |
| 37 | personal narratives as topic/ |
| 38 | 31 or 32 or 33 or 34 or 35 or 36 or 37 |
| 39 | (qualitative or (focus adj1 group*) or interview* or (mixed adj2 method*)).tw,kw. |
| 40 | ((experience* or narrative* or narration*) adj3 (nurse* or physician* or doctor* or (general adj2 practitioner*) or GP* or psychologist* or psychotherapist* or dietician* or dietitian* or nutritionist* or (occupational adj2 therapist*) or (physical adj2 therapist*) or physiotherapist* or (social adj2 worker*) or **(h**ealth adj2 (personnel or professional* or worker*)))).tw,kw. |
| 41 | 31 or 32 or 33 or 34 or 35 or 36 or 37 or 38 or 39 or 40 |
| 42 | 30 and 41 |
| 43 | exp Pulmonary Disease, Chronic Obstructive/ |
| 44 | (copd or (chronic adj2 obstructive adj2 (pulmonary adj2 disease))).tw,kw. |
| 45 | 43 or 44 |
| 46 | 42 and 45 |
| 47 | limit 46 to (comment or editorial or letter) |
| 48 | 46 not 47 |
| 49 | limit 48 to yr="2002 -Current" |
|  |  |
| **DIABETES** | Same search from 1- 42 |
| 43 | exp Diabetes Mellitus/ |
| 44 | (diabetes or diabetic*).tw,kw. |
| 45 | 43 or 44 |
| 46 | 42 and 45 |
| 47 | limit 46 to (comment or editorial or letter) |
| 48 | 46 not 47 |
| 49 | limit 48 to yr="2002 -Current" |
|  |  |
| **Chronic Kidney Disease** | Same search as before from 1 to 42 |
| 43 | exp Renal Insufficiency, Chronic/ |
| 44 | (chronic adj1 ((kidney adj1 (disease* or insufficienc*)) or (renal adj1 (disease* or insufficienc*)))).tw,kw. |
| 45 | 43 or 44 |
| 46 | 42 and 45 |
| 47 | limit 46 to (comment or editorial or letter) |
| 48 | 46 not 47 |
| 49 | limit 48 to yr="2002 -Current" |
|  |  |
