# Supplementary material for: Working with patients suffering from chronic diseases can be a balancing act for health care professionals - a meta-synthesis of qualitative studies
Source: BMC Health Serv Res. 2020 Feb 10;20:98. doi: 10.1186/s12913-019-4826-2 (PMC7011477; doi:10.1186/s12913-019-4826-2)
Supplement: Supplementary file 2 — Additional file 2. Detailed study appraisal. [file 12913_2019_4826_MOESM2_ESM.docx]

|  | **CASP** | | | | | | | | | | **COREQ** |
| --- | --- | --- | --- | --- | --- | --- | --- | --- | --- | --- | --- |
|  | **Section A** | | | | | | **Section B** | | | **Section C** |  |
|  | **Valid results?** | | **Worth continuing?** | | | | **What are the results?** | | | **Will the results help locally?** | **Methodological orientation and theory** |
|  | Clear statement of the aims? | Qualitative methodology appropriate? | Research design appropriate to address the aims? | Recruitment strategy appropriate to the aims? | Data collection addressing the research issue? | Considerations of the relationship between researcher and participants? | Considerations of ethical issues? | Sufficiently rigorous data analysis? | Clear statement of the findings? | Valuable research? |  |
| Brown et. al. 2013 | + | + | + | + | + | - | + | + | + | + | ? |
| Kim et. al. 2016 | + | + | + | + | + | - | + | + | + | + | + |
| Matthews and Tre-weth 2015 | + | + | + | ? | ? | - | + | ? | + | + | - |
| Pooley et. al. 2015 | + | + | ? | + | + | - | + | + | + | + | + |
| Tam-Tham et. al. 2016 | + | + | - | + | + | - | + | + | + | + | + |
| Tonkin-Crine et. al. 2015 | + | + | + | + | + | - | + | + | + | + | + |
| Walker et. al. 2012 | + | + | + | + | + | - | + | + | + | + | - |
| Crawford 2010 | + | + | + | + | + | + | + | + | + | + | + |
| Zakrisson and Hägglund 2010 | + | + | + | + | + | - | + | + | + | + | + |
| Risør et. al. 2013 | + | + | + | + | + | - | - | + | + | + | + |
| Wens et. al. 2005 | + | + | + | + | + | + | - | + | + | + | ? |
| Abdulhadi et. al. 2013 | + | + | + | ? | + | + | + | + | + | + | ? |
| Crowshoe et. al. 2017 | + | + | + | + | + | ? | + | + | + | + | - |
| Bostrom et. al. 2012 | + | + | + | + | + | ? | + | + | + | + | - |
| Sveningsson et. al. 2011 | + | + | + | + | + | + | + | + | + | + | + |
| Huber et. al. 2011 | + | + | + | + | + | ? | + | + | + | ? | - |
| Tierney et. al. 2017 | + | + | + | + | + | + | - | + | + | + | + |
| + yes, - no, ? unclear. | | | | | | | | | | | |
